# Trends in sodium–glucose transport protein 2 inhibitor prescriptions: application of the ATC/DDD system to NDB open data

**DOI:** 10.1007/s13340-026-00899-1

**Published:** 2026-04-22

**Authors:** Takefumi Miyake, Mai Hashimoto, Daisuke Kadowaki

**Affiliations:** 1 Division of Pharmacy, Omi Medical Center, 1660 Yabase-cho, Kusatsu City, Shiga 525-8585 Japan; 2 Department of Pharmacy, Omi Fureai Hospital, 1629-5 Yabase-cho, Kusatsu City, Shiga 525-0066 Japan; 3 Faculty of Pharmaceutical Sciences, Kobe Gakuin University Port Island, Campus 1-1-3 Minatojima, Chuo-ku, Kobe, Hyogo 650-8586 Japan; 4 Faculty of Pharmaceutical Sciences, Sojo University, 4-22-1 Ikeda, Nishi-ku, Kumamoto City, Kumamoto 860-0082 Japan

**Keywords:** Sodium–glucose co-transporter-2 inhibitors, NDB open data Japan, ATC/DDD system, Prescription trend, DDD per 1000 inhabitants per day

## Abstract

**Background:**

Understanding regional patterns of pharmaceutical use is essential for effective resource allocation and promoting rational drug use. The NDB Open Data of the Ministry of Health, Labor and Welfare offers a high-quality national database that comprehensively captures usage patterns. However, direct comparisons based on prescription volume are challenging owing to differences in drug formulations and dosages, and increasing generic drug use.

**Objective:**

This study examined sodium–glucose transport protein 2 inhibitor (SGLT2i) usage patterns across Japanese prefectures by comparing prescription quantities and drug costs using the NDB Open Data.

**Methods:**

Using the NDB Open Data, we extracted single-component SGLT2i formulations (pharmacological classification 396) and calculated the Defined Daily Dose (DDD) per 1000 inhabitants per day (DID) using WHO Anatomical Therapeutic Chemical (ATC) codes.

**Results:**

During the fiscal year 2022, urban prefectures, including Tokyo, Kanagawa, Osaka, Aichi, and Saitama, recorded the highest SGLT2i prescription quantities. Conversely, DID values were notably higher in rural areas such as Fukushima, Tochigi, Kagawa, Oita, and Iwate, indicating notable regional differences that could not be explained solely by population size. Annual prescriptions consistently increased across all drugs, reflecting the impact of expanded indications and product discontinuation. Canagliflozin demonstrated relatively low domestic usage in DID despite a similar number of tablets prescribed compared to other drugs, suggesting distinct regional prescription patterns.

**Conclusion:**

Combining the NDB Open Data with the ATC/DDD system identified regional SGLT2i usage. This approach could enable standardized comparisons within drug classes, providing invaluable evidence to support appropriate drug use guidelines and informed policy formulations.

## Introduction

**Fig. 1:**
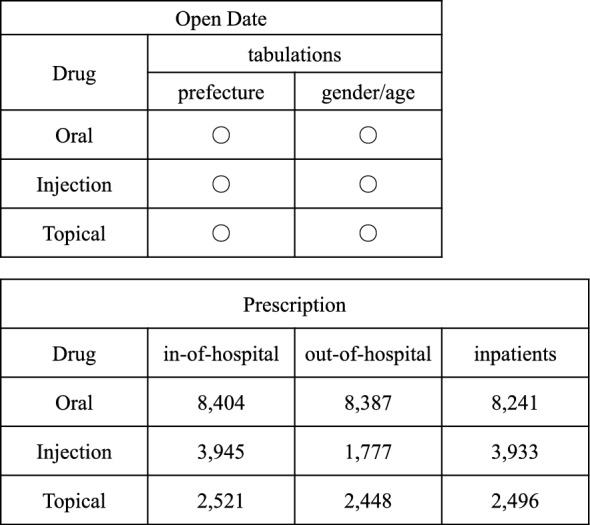
Published drug data and the number of aggregated items from the NDB Open Data. This figure presents drug-related data obtained from the NDB Open Data for the fiscal year (FY) 2022. NDB, National Database of Health Insurance Claims and Specific Health Checkups of Japan

Based on the “Act on Assurance of Medical Care for Elderly People,” the Ministry of Health, Labour and Welfare (MHLW) began building the “National Database of Health Insurance Claims and Specific Health Checkups of Japan (NDB)” in 2009. There are two types of NDB: NDB data that warrants payment and strict management, and open data that is available to the general public (NDB Open Data) [1]. Since October 2016, NDB Open Data has been provided annually with basic tabulations, and includes information regarding health insurance claim data (receipts) since fiscal year (FY) 2014 and information related to specific health checkups since FY2013 [2]. In this database, drug data are broadly classified by three dosage forms: oral, topical, and injection, and divided into prescription categories: outpatient (out-of-hospital and in-hospital) and inpatient (Fig. 1). The ranking of drugs with the highest prescription volume (e.g., tablet count) is published based on the standard unit of National Health Insurance (NHI) drug price standard listing for each drug category. In the first session, information on the top 30 drugs by annual drug prescription volume was released, and in the second through the eighth sessions, this was expanded to the top 100 drugs. The 9th edition published the top 100, 300, and 500 drugs by prescription volume for each drug category. The NDB Open Data provide information such as product name, unit (e.g., mg, mL, and tablet), drug price, and total (prescription volume), compiled by prefecture, sex, and age (every 5 years) according to the drug category. NDB and NDB Open Data have been extensively utilized [3–5]. Accordingly, these databases serve as invaluable sources of high-quality evidence on actual drug use on a national scale, with applications in medical information and pharmaceutical sciences. On the other hand, conventional methods for evaluating drug use predominantly rely on drug costs and the number of tablets prescribed, referred to as “prescription volume.” However, multiple product names for a single ingredient**—**including different specifications, formulations, and generics**—**complicate direct comparisons between drugs and across regions, especially when accounting for population and other factors. Therefore, as an indicator to evaluate drug use, the World Health Organization (WHO) recommends a drug code (the Anatomical Therapeutic Chemical [ATC]) comprising five levels assigned to each drug and the average daily maintenance dose (Defined Daily Dose [DDD]), published on the WHO Collaborating Centre (WHOCC) for Drug Statistics Methodology website [6]. The ATC/DDD system is widely used internationally as a standardized method for evaluating drug use [7–9]. In Japan, few studies have applied the ATC/DDD system as an evaluation criterion for statistical surveys on the use of drugs, including antidiabetic medications. We focused on sodium–glucose co-transporter-2 inhibitors (SGLT2is), which rapidly became the first-line treatment for diabetes after launch and have multiple indications, including chronic kidney disease and heart failure.

In the present study, we extracted the SGLT2i prescriptions by prefecture from the NDB Open Data for FY2015 to FY2022 and compared them in terms of the number of tablets and drug costs to identify regional differences. In addition, the ATC/DDD system was applied to analyze regional differences in SGLT2i usage across Japan, assessing the prescription volume and DDD per 1000 inhabitants per day (DID) by prefecture. This study was conducted to verify the further utilization of the data obtained by applying the ATC/DDD system.

## Methods

For pharmaceutical data, we used data from the 2nd (FY2015) to the 9th (FY2022) editions of the NDB Open Data, which are published on the MHLW website and list the top 100 prescription quantities for each three-digit drug classification [2]. The minimum aggregation unit was prescription volume: items with a total of fewer than 1000 were indicated with a “-” (hyphen). If only one item with a total of fewer than 1000 was hyphenated, the item other than the total was counted as “0” (zero) to identify the facility. Excel data were obtained from the quantities of outpatient (out-of-hospital and in-hospital) and inpatient drugs by prefecture, listed in “Prescription Drugs—Internal Drugs” in this dataset. Antidiabetic drugs (drug category number 396) were extracted, and SGLT2i in this category were included in the analysis (Table 1). However, only single-drug formulations were analyzed, excluding fixed-dose combinations. Although the 9th edition listed the top 300 antidiabetic drugs (Drug Classification Number 396), the top 100 drugs were counted to match the number of drugs in the 2nd and subsequent editions. Using the ATC/DDD system figures from the WHOCC website [6], each drug was counted by specification and ingredient by year. However, because some drugs were not listed in the ATC/DDD Index 2024, the ATC code for tofogliflozin was set to A10BK10, and the DDDs for ipragliflozin, luseogliflozin, and tofogliflozin were based on the daily dosages listed in the Japanese electronic package insert. We evaluated (1) the prescription volume of each drug by standard; (2) the drug cost, calculated by multiplying the number of prescriptions by the drug price; (3) the number of SGLT2i prescriptions per day per prefecture, (g)/DDD (g) divided by the population of the prefecture; and (4) DDDs/1,000 000 persons/day, multiplied by 1000 to correct for differences between the population and number of prescriptions by the SGLT2i standard (DID) [10]. The estimated population (as of October 1, 2022) from the Statistics Bureau of the Ministry of Internal Affairs and Communications website was used [11].

**Table 1:** A10BK sodium–glucose co-transporter 2 (SGLT2) inhibitors

ATC code	Name	DDD	Units	Adm.R
A10BK01	Dapagliflozin	10	mg	O
A10BK02	Canagliflozin	0.2	mg	O
A10BK03	Empagliflozin	17.5	mg	O
A10BK04	Ertugliflozin*	10	mg	O
A10BK05	Ipragliflozin	50**	mg	O
A10BK06	Sotagliflozin*			
A10BK07	Luseogliflozin	2.5**	mg	O
A10BK08	Bexagliflozin*			
A10BK09	Enavogliflozin*			
A10BK10**	Tofogliflozin**	20**	mg	O

Quoted from ATC/DDD Index 2024

^*^Not yet available in Japan,** Not listed in ATC/DDD Index 2024

*ATC* Anatomical Therapeutic Chemical, *DDD* Defined Daily Dose, *Adm.R* Route of administration

## Results

**Fig. 2:**
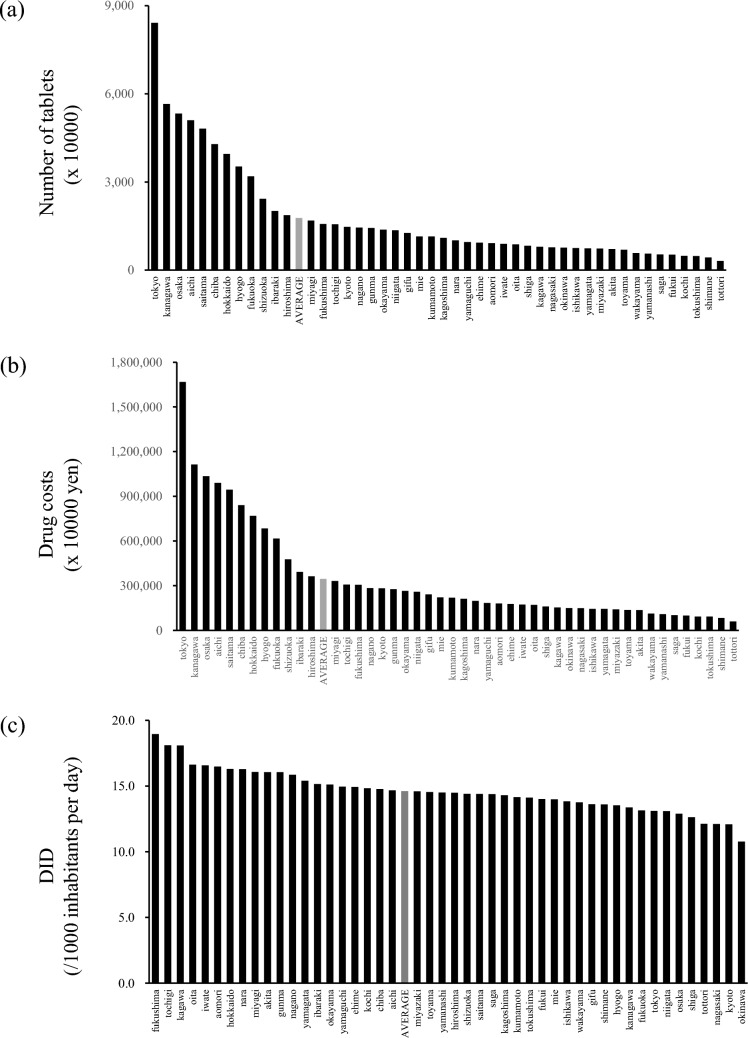
Prefecture-specific prescription volume of SGLT2 inhibitors in FY2022. The prescription volume of SGLT2 inhibitors in each prefecture for FY2022 was analyzed using **a** the number of tablets, **b** the number of tablets multiplied by drug price, and **c** DID. FY, fiscal year; SGLT2, sodium–glucose transport protein 2

**Fig. 3:**
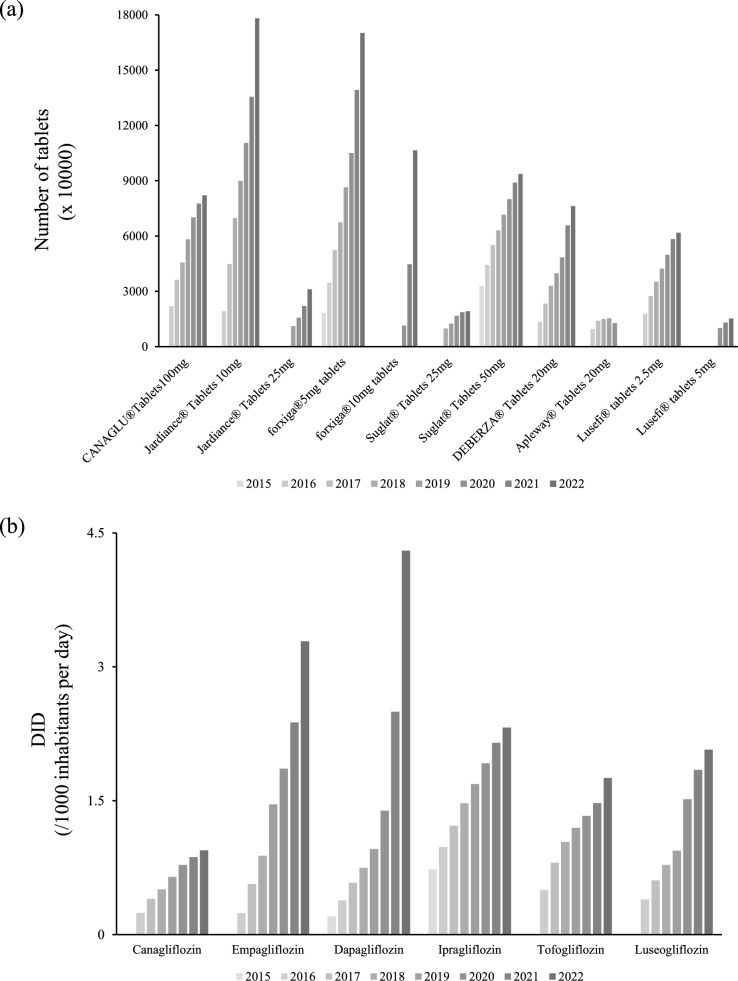
Annual trends in the prescription volume of SGLT2 inhibitors. The prescription volume of SGLT2 inhibitors from fiscal year (FY) 2015 to FY2022 was analyzed based on **a** the number of tablets per product specification and **b** the DID for each active ingredient. DID, Defined Daily Dose per 1000 inhabitants per day; SGLT2i, sodium glucose co-transporter 2 inhibitor

The SGLT2i prescription volume by prefecture for FY2022 is illustrated in Fig. 2a. The average prescription volume was 17,751,046.5, with Tokyo (1st), Kanagawa (2nd), Osaka (3rd), Aichi (4th), and Saitama (5th) ranking highest, and Tottori (47th), Shimane (46th), Tokushima (45th), Kochi (44th), and Fukui (43rd) ranking lowest. Twelve prefectures exceeded this average. Drug costs, calculated using FY2022 drug prices, are shown in Fig. 2b. The average drug cost was ¥3,460,536,849.1, with 12 prefectures exceeding the average cost. The top five prefectures were Tokyo (1st), Kanagawa (2nd), Osaka (3rd), Aichi (4th), and Saitama (5th), while the bottom five were Tottori (47th), Shimane (46th), Tokushima (45th), Kochi (44th), and Fukui (43rd). Furthermore, the DID was used to adjust for differences in prescription volume due to factors such as population and drug specifications (Fig. 2c). According to the DID rankings, Fukushima ranked first, followed by Tochigi, Kagawa, Oita, and Iwate, while Okinawa (47th), Kyoto (46th), Nagasaki (45th), Tottori (44th), and Shiga (43rd) ranked the lowest. Although comparing prescription volumes across prefectures revealed differences influenced by population, this effect was offset by adjusting for DID. Next, we analyzed the prescription volume according to drug specifications and year. Excluding Apleway®, which was withdrawn from the market, prescription volumes increased annually for all drugs. However, some drugs showed significant increases in prescription volumes, allowing the identification of prescription trends related to expanded indications or market withdrawals (Fig. 3a). Next, we calculated annual trends using DID (Fig. 3b). A comparison of prescription quantities by the number of tablets showed that canagliflozin had similar prescription quantities to ipragliflozin, tofogliflozin, and luseogliflozin. However, when compared using DID, canagliflozin tended to be prescribed less than other SGLT2i in Japan.

## Discussion

In the present study, we identified regional differences in the use of SGLT2i across Japan. High prescription volumes were observed in urban areas, such as Tokyo, Kanagawa, and Osaka, suggesting that population size and the availability of medical resources may be contributing factors. However, when evaluated using DID, notable prescription levels were also seen in regional cities such as Fukushima, Kagawa, and Tochigi, suggesting that medical care may be tailored to regional characteristics and patient needs. Standard measures are required to evaluate the appropriate and rational use of pharmaceuticals [12]. Traditionally, these measures have been based on the quantity of drugs prescribed (e.g., number of tablets and grams) or drug costs, depending on the drug price. General names of pharmaceuticals (Japanese Accepted Names) may have multiple brand names or specifications, owing to generic or co-marketed drugs. Additionally, when comparing drugs with the same therapeutic effect, the daily dosage and administration vary by drug, making it difficult to evaluate them appropriately by simply summing the prescribed quantities (e.g., number of tablets or grams).

In 2016, Japan formulated an “Action Plan for Antimicrobial Resistance (AMR) Countermeasures” to promote appropriate antimicrobial use by comparing trends in drug-resistant bacteria and the use of antimicrobial agents with those in other countries that have different background factors, such as medical expenditures and the number of hospital beds [11]. The dosage and administration of antimicrobial agents vary depending on the drug, and there are multiple standards. Therefore, it is not possible to compare the use of different types of antimicrobial agents by simply adding the total number of units or potencies [12]. To resolve these issues, DID—a drug use index—was calculated using the WHO ATC/DDD system [6]. For example, the amount of antimicrobial drugs used in Japan in 2013 (15.8 DID) was higher than in 2009 (14.7 DID), and the amount of both oral and injectable drugs used increased progressively. However, no significant differences were observed compared with the European Union (21.6 DID, 2014), South Korea (21.7 DID, 2012), or the United States (24.9 DID, 2014) [12]. As the ATC/DDD system is used globally to monitor the use of pharmaceuticals [7–9], the results of the present study suggest that DID—used in several countries other than Japan—should be adopted as the standard to evaluate the appropriate use of pharmaceuticals in the future, rather than relying on the number of prescriptions.

Because trends in drug costs resemble those in the number of prescriptions, either monetary values or prescription counts can be used as standards, depending on the objective (Fig. 2a, b). Drug costs are often used as standards because they provide valuable information to those involved in the development, production, distribution, and sales of pharmaceuticals. However, in Japan, drug prices are reduced every two years. This makes simple international comparisons difficult, as drug prices differ depending on the country or region [12–14].

Trends, such as the introduction of new specifications and discontinuation of drugs, can be identified from the prescription quantities for each drug specification and year (Fig. 3a). Furthermore, when considering the annual trends by DID, we found that canagliflozin tended to be prescribed less than other SGLT2is (Fig. 3b). Inoue et al. revealed that the increase in the annual prescription volume of all SGLT2is, excluding luseogliflozin, was greater in areas with a high number of cardiologists. The authors also found that the number of areas with cardiologists was positively associated with an increase in the prescription volume of SGLT2is [13–15]. Accordingly, in addition to drug characteristics, regional characteristics—such as the number of specialists for specific diseases—may also affect the choice of prescribing canagliflozin. Additionally, the lower DID of Canagliflozin compared to other SGLT2 inhibitors in this study may be influenced by its combination products with DPP-4 inhibitors (Canalia®). In Japan, combination therapy with DPP-4 inhibitors is common, and the use of fixed-dose combinations is known to be high. Indeed, analysis of NDB open data (FY2022) showed that the prescription volume ratio of Empagliflozin (Jardiance®) to TradianceAP® was approximately 3: 1, and Ipragliflozin (Suglat®) and Sujanu® was approximately 1.5:1. In contrast, the ratio of Canagliflozin (Canaglu®) and Canalia® was approximately 1.1:1, indicating a relatively higher proportion of combination products (data not shown). Therefore, the low DID for Canagliflozin monotherapy likely reflects a clinical trend toward increased switching to combination drugs rather than necessarily indicating low actual usage frequency.

As mentioned above, although there are no regional differences in Japan, the NHI drug price is revised every two years; therefore, the level of drug costs and the NHI drug price itself may change annually, potentially impacting the results of long-term studies. The term “prescription volume” refers to the amount of medicine, such as the number of tablets or grams, or the number of times the medicine is used. However, this measure is not always appropriate because results can vary depending on the potency (in the case of drug weight) or the amount of ingredients per tablet (in the case of tablet number). Given that DID is applied on a regional and national basis, it is useful for comparisons within a region or country. Unlike the number of drugs, DID does not account for differences in drug form; therefore, it is less susceptible to factors such as changes in the Japanese population and is valuable for observing trends over time [6].

The analysis combining the NDB open data used in this study with the ATC/DDD method demonstrates one approach for understanding drug usage trends on a national scale. However, with the increasing prevalence of generic drugs and combination drugs, careful interpretation considering DDD settings and drug classification will be required in the future.

### Limitations

Several factors should be considered when interpreting prescribing volumes and regional differences for SGLT2 inhibitors. The NDB Open Data is not linked to patient information; therefore, it is not possible to conduct individualized or segmented analysis. Drug utilization is influenced by multifactorial elements, including not only differences in therapeutic classes but also time to market, accumulation of evidence, expanded indications, drug pricing, prescribers' experience, and the pharmacological properties of each drug [16]. These factors may have contributed to differences in prescribing volumes and DID.

Furthermore, when evaluating DID differences by prefecture, the impact of regional population composition (gender and age groups) must be considered. SGLT2 inhibitors have been reported to be prescribed more frequently in males and relatively younger age groups [17], suggesting that differences in regional gender and age distributions may contribute to regional DID variations. Indeed, analysis of the 2022 NDB data showed that prescription volume peaked for both men and women in the 70–74 age group. However, while prescriptions exceeded 85 million for men, they remained at 50 million for women. Overall, prescriptions for those under 65 accounted for 43% of the total, while prescriptions for those aged 65 and over accounted for 57%, indicating a higher prescription rate among the elderly (data not shown). Because the NDB open data used in this study aggregates prescription volume by drug separately for age/gender and prefecture, it was difficult to conduct an analysis adjusting prefecture-level DID for age and gender. The results of this study should be interpreted as descriptive epidemiological analysis without age and gender adjustment. Future analyses should combine detailed NDB open data with other epidemiological datasets to perform adjustments for diabetes prevalence and population composition.

Although an NDB database containing more detailed information is available, strict data management requirements and the associated charge are limiting factors that will be considered in future investigations. However, as demonstrated in this study, valuable insights into the situation in each prefecture can be obtained from the NDB public data. In addition, to resolve regional disparities in medical care, information must be shared between prefectures and between facilities. Therefore, the model used in the previous survey on antimicrobial use could be applied to the treatment of various diseases, including diabetes.

## Conclusion

The findings of this study suggest that the ATC/DDD system is useful for evaluating SGLT2i in Japan, including regional differences and annual trends. In future studies, we plan to comprehensively analyze the factors impacting regional differences and drug selection, evaluate diabetes drugs using the ATC/DDD system, and verify how to provide optimal medical care in each region.

## Data Availability

The data generated and analyzed during the current study are available from the corresponding author upon reasonable request.
